# Electrochemical and Electroconductive Behavior of Silk Fibroin Electrospun Membrane Coated with Gold or Silver Nanoparticles

**DOI:** 10.3390/membranes12111154

**Published:** 2022-11-16

**Authors:** Wilson Agudelo, Yuliet Montoya, Alejandra Garcia-Garcia, Adriana Restrepo-Osorio, John Bustamante

**Affiliations:** 1 Grupo de Dinámica Cardiovascular, Línea Ingeniería de Tejidos y Protésica Cardiovascular, Universidad Pontificia Bolivariana, Medellín 050031, Colombia; 2 Comité de Trabajo de Bioingeniería Cardiovascular, Sociedad Colombiana de Cardiología y Cirugía Cardiovascular, Bogotá 110121, Colombia; 3 Grupo de Síntesis y Modificación de Nanoestructuras y Materiales Bidimensionales, Centro de Investigación en Materiales Avanzados S.C., Parque PIIT, Km 10, Autopista Monterrey-Aeropuerto, Apodaca 66628, Mexico; 4 Grupo de Investigación sobre Nuevos Materiales, Universidad Pontificia Bolivariana, Medellín 050031, Colombia

**Keywords:** electrochemical impedance spectroscopy, silk fibroin, metal nanoparticles, electrospun membranes, impregnation coatings

## Abstract

The surface modification of materials obtained from natural polymers, such as silk fibroin with metal nanoparticles that exhibit intrinsic electrical characteristics, allows the obtaining of biocomposite materials capable of favoring the propagation and conduction of electrical impulses, acting as communicating structures in electrically isolated areas. On that basis, this investigation determined the electrochemical and electroconductive behavior through electrochemical impedance spectroscopy of a silk fibroin electrospun membrane from silk fibrous waste functionalized with gold or silver nanoparticles synthetized by green chemical reduction methodologies. Based on the results obtained, we found that silk fibroin from silk fibrous waste (SF_w_) favored the formation of gold (AuNPs-SF_w_) and silver (AgNPs-SF_w_) nanoparticles, acting as a reducing agent and surfactant, forming a micellar structure around the individual nanoparticle. Moreover, different electrospinning conditions influenced the morphological properties of the fibers, in the presence or absence of beads and the amount of sample collected. Furthermore, treated SF_w_ electrospun membranes, functionalized with AuNPs-SF_w_ or AgNPS-SF_w_, allowed the conduction of electrical stimuli, acting as stimulators and modulators of electric current.

## 1. Introduction

The recent integration of nanotechnology into the biomedical field has made it possible to make use of the physicochemical properties inherent to materials on a nanometric scale to propose new therapeutic methodologies, such as the controlled release of drugs [1], tissue repair [2], techniques for cell diagnosis, and therapy [3]. Moreover, it is necessary to carry out processes that allow the obtaining of nanostructures with controlled size and shape, high purity in environmentally friendly conditions, and with low levels of toxicity [2,3]. To achieve this, the use of functionalization processes is required, generally with elements of natural origin, giving rise to biocomposite materials.

In the search for a biomaterial that mimics functional behavior and has a favorable biocompatible response, different polymers of natural origin have been studied, among which is silk fibroin (SF), a protein extracted from different families of silkworms such as Bombyx mori and of some arthropods such as spiders. This protein has exhibited biocompatibility, controllable biodegradability, and superior mechanical properties to those of other natural biopolymers such as collagen [4,5], so SF is considered a material of interest for use in the field of tissue engineering. Moreover, it can be electrospun under specific conditions.

Nowadays, the incorporation of inorganic nanoparticles with biocompatible and electroconductive characteristics in the functionalization of materials with application to tissue engineering seeks to provide controllable electrical properties that do not interfere with the electrical conductance of the action potential, and that in turn allows for modulating the geometry and topography of the material to mimic the morphological characteristics of the native extracellular matrix [6,7]. In view of this, it is necessary to carry out processes that allow obtaining nanostructures with controlled size and shape, high purity, in environmentally friendly conditions, and with low levels of cytotoxicity [2,3]. To achieve this, the use of functionalization processes is required, generally with elements of natural origin, giving rise to biocomposite materials [8,9].

On the other hand, to determine the electro-conductive characteristics of biocomposite materials, several techniques can be used, among which is electrochemical impedance spectroscopy, which allows the electrical properties of materials to be analyzed at different conditions, obtaining frequency spectra of impedance, conductivity, and permittivity. These are adjusted by mathematical models that allow for elucidating the electrical behavior (resistor, inductor, capacitor, constant phase elements, among others) [10].

In this context, and in order to propose an electrospun membrane that exhibits electrochemical and electroconductive characteristics, the research purpose was the development of a biocomposite electrospun membrane with electro-conductive properties based on a natural polymeric structure of silk fibroin from silk fibrous waste, coated with gold or silver nanoparticles, which were synthesized from a chemical reduction with the same SF_w_ in one step. This biocomposite could be used as a therapeutic strategy for tissue engineering, like an electroconductive scaffold that facilitates ionic interaction between isolated electric areas.

## 2. Materials and Methods

For this investigation, gold trichloride hydrochloride (HAuCl_4_ 3H_2_O), lithium bromide (LiBr, ReagentPlus, ≥99%), sodium carbonate (Na_2_CO_3_, ReagentPlus), and polyethylene oxide (PEO, Mv ~ 900,000) were obtained from (Sigma Aldrich, St. Louis, MO, USA). Sodium hydroxide (NaOH pellets), methanol for analysis (EMSURE ACS, ISO, Reag. Ph Eur), potassium chloride (EMSURE), potassium dihydrogen phosphate (EMSURE ISO), sodium phosphate, dibasic, heptahydrate (EMSURE ACS), and sodium bicarbonate (EMSURE ISO) were obtained from (Merck Millipore, Darmstadt, Germany). Silver nitrate (AgNO_3_ pure, pharma grade) and sodium chloride (NaCl pure, pharma grade) were obtained from (PanReac Applichem, Darmstadt, Germany). The NIH/3T3 fibroblast cell line was purchased from (American Type Culture Collection (ATCC), Manassas, VA, USA) and Dulbecco’s Modified Eagle’s Medium was obtained from (Lonza Bioscience, Durham, NC, USA). Fetal bovine serum was purchased from Microgen, trypan blue was obtained from (Loba Chemie Mumbai, India), and 3-(4,5-dimethylthiazol-2-yl)-2,5-diphenyltetrazolium bromide (MTT) was acquired from (Alfa Aesar, Haverhill, MA, USA).

### 2.1. Silk Fibroin from Silk Fibrous Waste Extraction (SF_w_)

Silk fibrous wastes from the Bombyx mori silkworm acquired from Corporación para el Desarrollo de la Sericultura del Cauca—CORSEDA (Popayán, Cauca, Colombia) were used. The silk fibers were degummed by immersion in aqueous Na_2_CO_3_ solution. Then, it was dissolved in an aqueous LiBr solution and dialyzed until reaching a stable conductivity, following the protocol reported by Jaramillo-Quiceno et al. [11].

### 2.2. Synthesis of Gold and Silver Nanoparticles

SF_w_ to 0.5% *v*/*v* was mixed with each of the precursor solutions of 2.5 mM HAuCl_4_ and 10 mg/mL AgNO_3_. Then, each of the solutions was incubated in light at 60 W for 24 h.

### 2.3. Preparation and Treatment of Silk Fibroin Electrospun Membranes

A SF_w_/PEO homogeneous solution was electrospun using a flow of 0.7 mL/h and voltage of 16 kV, volumetric ratio of SF_w_:PEO of 50:50, and a needle-collector distance between 10 cm to 25 cm, with a flat plate type static collector. Then, electrospun SF_w_/PEO membranes were treated in methanol for 10 min and 15 min and taken under vacuum for 24 h to promote the evaporation of the solvent. Finally, they were washed with deionized water at 37 °C for 48 h to remove the PEO, and finally, they were dried at room temperature for 24 h.

### 2.4. Impregnation Coatings of Treated Electrospun Membranes with AuNPs-SF_w_ o AgNPs-SF_w_

Treated membranes were coated with AuNPs-SF_w_ or AgNPs-SF_w_ solutions by impregnation coatings for periods of 4 h and 24 h. Then, one sample for each immersion period and type of nanoparticle was washed with deionized water, while the other samples were not washed. Finally, all membranes were dried at room temperature.

### 2.5. UV-Visible Spectrophotometry and FTIR Spectroscopy Analysis

Gold and silver nanoparticles, as well as the treated and functionalized membranes, were analyzed in a UV-visible spectrophotometer (UV-Vis-NIR Cary 5000, Agilent Technologies, Santa Clara, CA, USA) and (Lambda Bio 10, Perkin Elmer, Waltham, MA, USA) in a wavelength range between (1100–200) nm. As controls, 0.5% SF_w_ solutions and membranes treated without functionalizing with the nanoparticles were used. To determine changes in the functional groups of untreated, treated, and functionalized membranes, a (Nicolet iS50 FTIR-ATR spectrometer, Thermo Fisher Scientific, Waltham, MA, USA) at a resolution of 4 cm^−1^ and 32 scans was used.

### 2.6. Scanning Electron (SEM) and Field Emission (FESEM) Microscopy Analysis

Gold and silver nanoparticles, untreated, treated, and functionalized membranes were analyzed on a (Nova NanoSEM 200, Hillsboro, OR, USA) field emission scanning electron microscope and on a scanning electron microscope (NeoScope JCM-6000 Plus, Jeol, Akishima, Japan) operated at 15 kV. The micrographs were analyzed with Fiji^®^ software developed by Schindelin et al. [12] for the diameters and distribution histograms’ particles and fibers.

### 2.7. Electrochemical Impedance Spectroscopy Analysis

The electrochemical impedance spectroscopy (EIS) configuration consisted of an arrangement of three electrodes where the electrospun membranes were arranged on a graphite bar, which acted as a working electrode (WE_G_) with an exposed area of 5.8 cm^2^. Moreover, a graphite rod with a diameter of 5.2 mm was used as a counter electrode (CE_G_), an Ag/AgCl electrode as a reference (RE) and Hank’s Balanced Salt solution as a working solution. For linear sweep voltammetry measurements, a potentiostat (Ivium CompactStat.h, Ivium Technologies BV, Eindhoven, Netherlands) with scanning rates of 100 mV/s in a potential range between (0–1) V was used, using an Ag/AgCl reference electrode with saturated KCl solution. For electrochemical impedance measurements, these were performed at a sweep frequency of 1 × 10^6^ Hz to 0.1 Hz with 10 points for each decade, with an AC potential with 0.08 V amplitude and a range of DC potentials between 0 V and 1 V with a step of 400 mV between measurements.

### 2.8. MTT Assay

The cytotoxicity of the membranes without or functionalized with the gold or silver nanoparticles was measured with the MTT test, which allows for determining cell viability based on the mitochondrial activity of the cells from the direct interaction with the membranes. For this assay, 4.5 × 10^3^ cells/well of 3T3 fibroblasts were seeded in 96-well plates. Then, the membranes interacted with the 3T3 fibroblasts for 24 h. After MTT was added during (4–5) h, then it was treated with isopropanol to dissolve the formazan crystals. Later, it was left in incubation at 37 °C and the absorbance was measured at 570 nm using a spectrophotometer microplate reader (Multiskan FC, Thermo Fisher Scientific, Waltham, MA, USA).

### 2.9. Statistical Analysis

The synthesis of the gold and silver nanoparticles, the UV-visible measurements, and the electrochemical impedance spectroscopy were performed in triplicate at three independent times. In addition, a multivariate statistical analysis was performed using the Statgraphics Centurion XVI software (Statgraphics Technologies, The plains, VA, USA) which allowed obtaining the population mean, the standard deviation, and the 95% confidence intervals. For the statistical analysis of the particle and fiber size of the SEM and STEM micrographs, the Fiji^®^ software was used, obtaining the mean of the population, the standard deviation, and the 95% confidence intervals.

## 3. Results and Discussion

### 3.1. Synthesis of Gold and Silver Nanoparticles

**Figure 1:**
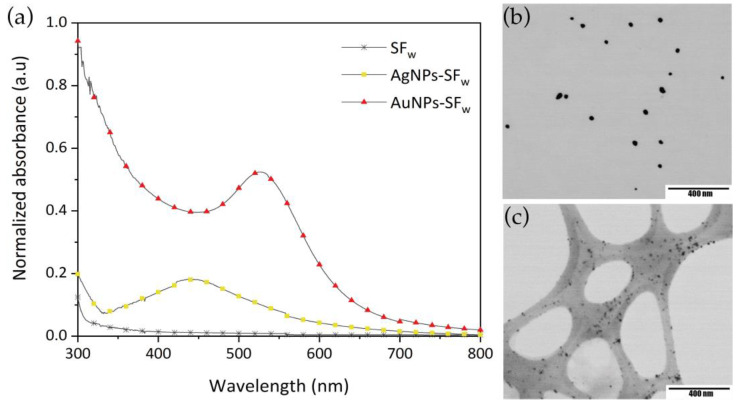
(**a**) Normalized UV-Vis absorption spectra of gold (line with red triangles) and silver (line with yellow squares) nanoparticles. STEM micrographs of (**b**) AuNPs-SF_w_ and (**c**) AgNPs-SF_w_. Scale bar, 400 nm.

In the analysis by UV-Vis spectrophotometry (Figure 1a), the presence of absorption bands corresponding to the surface plasmon resonance (SPR) of each metal ion at synthesized solutions were located at wavelengths of 526 nm for AuNPs-SF_w_ and 439 nm for AgNPs-SF_w_.

The presence of tyrosine residuals in the SF_w_ structure are responsible for the process of formation of gold and silver nanoparticles, which, based on the pH conditions in which the synthesis reaction takes place, establishes the mechanisms of particle formation and stability. In the case of gold nanoparticles, when presenting pH adjustment, the reaction mechanism is mediated by the electrons transfer through the deprotonation of oxygen and the formation of tyrosinase ion. However, for silver nanoparticles, as they do not present a pH adjustment, a reaction mediated by the transfer of electrons coupled to protons is generated [13,14,15,16].

From the analysis of the STEM micrographs of the metal nanoparticles (Figure 1b,c), it was found that the AuNPs-SF_w_ present particles with an average size (23 ± 6) nm, while AgNPs-SF_w_ exhibited average sizes of (14 ± 8) nm. The changes in particle size for each type of nanostructure are related to the final value of the pH and the concentration of the reducing agent, which in each case results in the reaction pathway that each synthesis follows and the way in which gold or silver ions are coupled in the nucleation and growth processes [15,17,18].

### 3.2. Preparation of Silk Fibroin Electrospun Membranes

**Figure 2:**
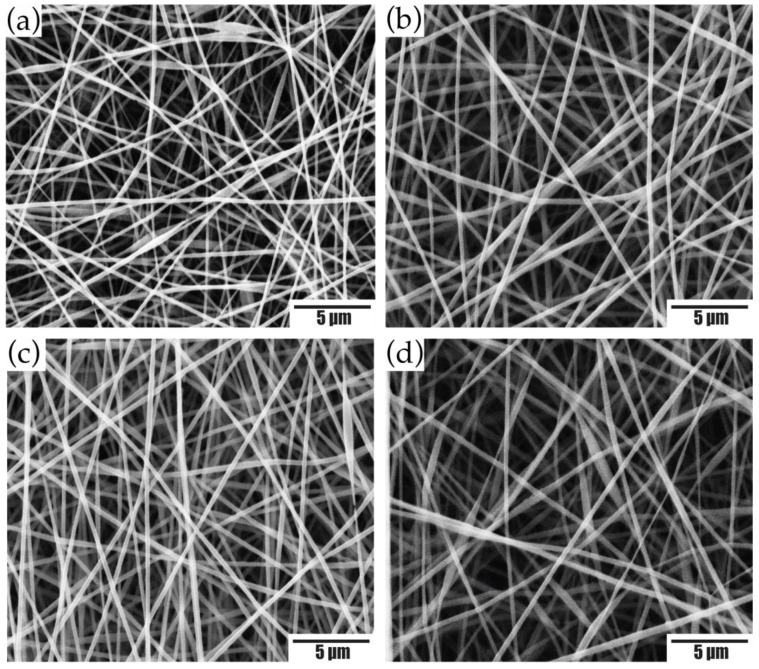
SEM micrographs of SFw/PEO electrospun membranes obtained at a volumetric ratio of 50:50, at distances of (**a**) 10 cm; (**b**) 15 cm; (**c**) 20 cm; (**d**) 25 cm. Scale bar, 5 μm.

To obtain the silk fibroin electrospun membranes, PEO was used as a modifying element of the electrospinning conditions [19]. From the results obtained, for the 50:50 SF_w_/PEO ratio (Figure 2a–d), the decrease in protein concentration caused an increase in viscosity that favored the polymer chains to overcome the surface tension, resulting in uniform fibers without the presence of defects [20].

The effect of the collection distance was evaluated. The membranes obtained at 50:50 SF_w_/PEO had fiber sizes of (194 ± 39) nm, (296 ± 47) nm, (264 ± 30) nm, and (281 ± 57) nm for distances of 10 cm, 15 cm, 20 cm, and 25 cm, respectively.

Furthermore, it was observed that the electrospinning distance has an influence on the sample collection area, where at distances of 10 cm and 15 cm, a circular geometry was observed without completely covering the collector, while, at distances of 20 cm and 25 cm, the collector was completely covered. In view of this, the difference in the collection area of the fibers is related to the projection of these on the collector, which decreases as the distance between the tip of the needle and the substrate decreases, forming a circular collection area [21].

According to the results, SF_w_/PEO electrospun membranes at a volumetric ratio of 50:50 and (20–25) cm were used, conditions that favored obtaining membranes with continuous fibers, without beads and a larger collection area.

### 3.3. Treatment of Electrospun Membranes

The use of organic solvents, such as ethanol and methanol as treatments in the silk fibroin electrospun membranes, reduces the percentage of non-crystalline structures of the amorphous phase present in the protein and decrease the solubility of the membrane in an aqueous medium. The use of these solvents favors the transition of fibroin macromolecules and their non-crystalline secondary structures to crystalline ones in the form of β-bends, increasing the crystalline phase of the membranes and making them hydrophobic [22,23].

**Figure 3:**
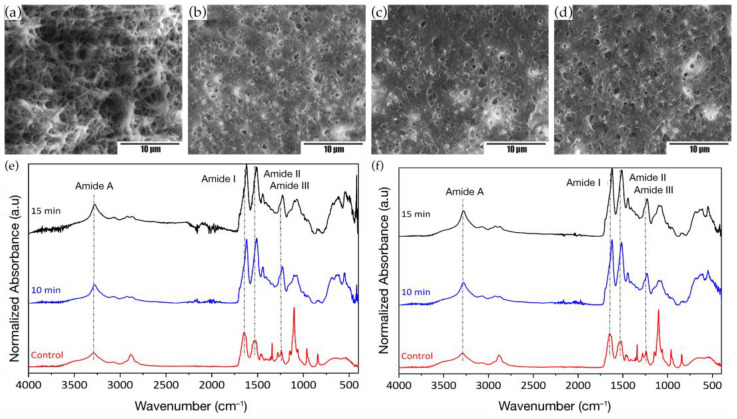
SEM micrographs of SF_w_/PEO electrospun membranes at a volumetric ratio of 50:50 treated with methanol at distances of 20 cm, for (**a**) 10 min; (**b**) 15 min. The distance of 25 cm, during (**c**) 10 min; (**d**) 15 min. Scale bar, 10 μm. Infrared absorption spectra of electrospun SF_w_/PEO membranes at a volumetric ratio of 50:50 treated with methanol for 10 min and 15 min. Where (**e**) 20 cm; (**f**) 25 cm. Untreated SF_w_/PEO electrospun membranes were used as controls.

To avoid the loss of material due to the solubilization of the membranes in aqueous media, the dehydration due to long periods of immersion, and remove the higher content of synthetic polymer, SF_w_/PEO electrospun membranes at a volumetric ratio of 50:50 and a distance of (20–25) cm were treated in methanol immersion for periods of 10 min and 15 min with subsequent washing in deionized water for 48 h. Based on the results obtained, changes in fiber sizes and rough morphologies were evidenced in the micrographs (Figure 3a–d), caused by the phase separation between SF_w_ and PEO extraction [24]. It was observed that the apparent porosity of the membranes is reduced by the increase in the size and thickening of the fibers, which could contribute to greater connectivity between fibers.

Concerning the functional changes that the electrospun membranes suffered from methanol, the analysis by infrared spectroscopy was performed, which were compared with the control spectrum of pristine SF_w_ and membranes without treatment. From the results obtained (see Figure 3e–f), it was found that the SF_w_ spectrum showed the characteristic peaks of the vibrational modes of the amides A (N-H stretching), I (C=O stretching), II (N-H bending and C-N stretching), and III (C-C stretch, C-N, and C-H bend) at wavenumbers of 3274 cm^−1^, 1635 cm^−1^, 1515 cm^−1^, and 1230 cm^−1^, respectively. In the untreated SF_w_/PEO membranes, these bands were located at wavenumbers of 3282 cm^−1^, 1640 cm^−1^, 1528 cm^−1^, and 1241 cm^−1^, which could indicate that electrospinning processes enhance the ability of the protein to self-organize its structure, similarly to native silk I (Table 1) [24].

Concerning the bands located at wavenumbers at 1100 cm^−1^, 962 cm^−1^, and 841 cm^−1^ in the absorption spectrum of the untreated SF_w_/PEO electrospun membranes, these corresponded to the stretching vibrations of the CO and bending of the CH out of the plane for polyethylene oxide [25,26], which after washing reduced in absorption intensity, indicating the removal of PEO from the electrospun membrane.

Regarding the changes in the fibroin structure, it was observed in the infrared absorption spectra of the treated electrospun membranes with methanol, the displacement of the bands associated with the characteristic vibrational modes of the protein, which were located at ~1620 cm^−1^, ~1511 cm^−1^, and 1228 cm^−1^ (Table 1). These displacements indicate the transition of the structures corresponding to random spirals to secondary structures in the form of β-sheets and β-turns due to the rearrangement induced by methanol to the SF_w_ chains and the formation of different hydrogen bonds [27,28].

Regarding the difference in the percentage of secondary crystalline structures in the form of β-sheets and β-turns varying the treatment times with methanol, it was found that immersion periods of 15 min increased the number of crystalline structures for both membrane types compared to being treated at 10 min immersion periods and untreated controls (Table 2).

Based on the above, it was found when that performing treatments with organic solvents such as methanol, the crystalline phase of SF_w_ electrospun membranes increases due to the transition of macromolecules and non-crystalline to crystalline secondary structures, favoring the membrane’s hydrophobicity with aqueous solutions [22,23].

### 3.4. Functionalization of Treated Membranes with Metal Nanoparticles

For the functionalization of treated membranes with gold or silver nanoparticles using impregnation coating, the membranes treated with methanol for 15 min were used, since these were those that had a higher content of crystalline structures. The samples were immersed in solutions with AuNPs-SF_w_ and AgNPs-SF_w_ for 4 h and 24 h. From the obtained results, a change in color of the membranes was evidenced, going from white to an intense red in the case of those functionalized with AuNPs-SF_w_ and to a light yellow-brown for the membranes functionalized with AgNPs-SF_w_. This change in tonality is related to the coupling of the different nanostructures on the surface of the electrospun material.

**Figure 4:**
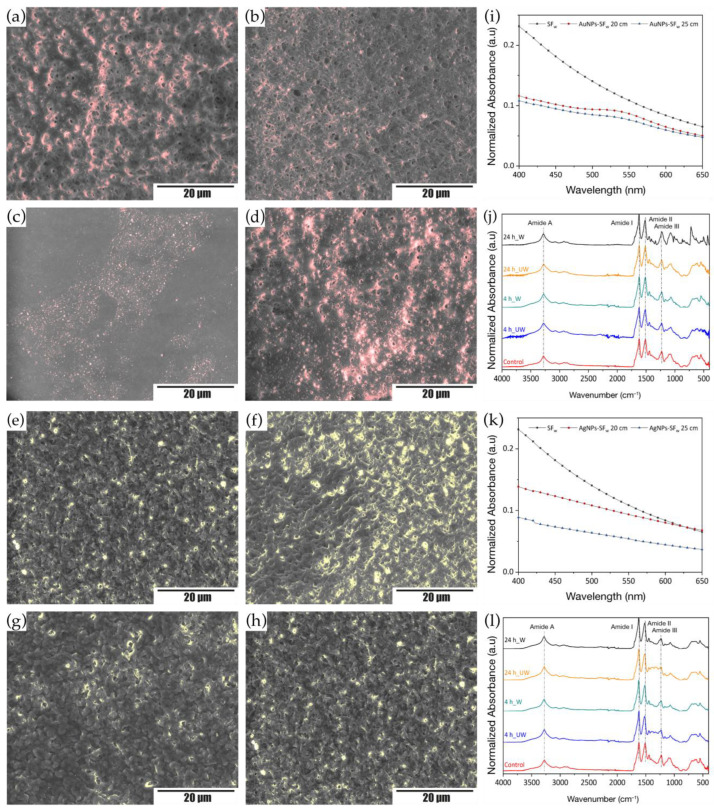
STEM micrographs of treated SF_w_ membranes and functionalized by impregnation coating for 4 h and 24 h with and without washing. Where (**a**,**b**) with AuNPs-SF_w_ with washing; (**c**,**d**) with AuNPs-SF_w_ without washing; (**e**,**f**) with AgNPs-SF_w_ with washing; (**g**,**h**) with AgNPs-SF_w_ without washing. Scale bar, 20 μm. UV-Visible absorption spectra of treated and functionalized SF_w_ membranes. Where (**i**) AuNPs-SF_w_ and (**k**) AgNPs-SF_w_. A SF_w_ membrane without impregnation coating was used as a control. FTIR-ATR absorption spectra obtained from treated and functionalized SF_w_ electrospun membranes. Where (**j**) AuNPs-SF_w_ and (**l**) AgNPs-SF_w_. Non-functionalized treated SF_w_ membranes were used as controls. Where UW—unwashed and W—washed.

Analysis of the unwashed membranes was performed by field emission microscopy, where the micrographs showed that the membranes functionalized with AuNPs-SF_w_ or AgNPs-SF_w_ without washing after the interaction time presented a greater deposition of nanoparticles in the fibers, in comparison with the membranes that were washed after the exposure time with the metal nanoparticles, which was evidenced by the presence of bright areas, red for the AuNPs-SF_w_, and yellow for the AgNPs-SF_w_ (Figure 4a–h). Such behavior is due to the solubility of AuNPs-SF_w_ or AgNPs-SF_w_ in aqueous solvents, which, when washed after the functionalization process, were removed from the membrane surface. On the other hand, and related to the functionalization times, it was evidenced that at longer immersion times there was a greater deposition of nanoparticles on the surface of the treated membranes, which could be related to a favoring of the coupling nanoparticle-membrane product of electrostatic bonding between amino groups present on the surface of electrospun membranes and functional groups exposed in the coating of gold and silver nanostructures by silk fibroin [29].

Moreover, the analysis by UV-Vis spectrophotometry of the SF_w_ membranes functionalized with the gold or silver nanoparticles was performed to detect the characteristic resonance plasmon for each type of nanostructure present on the surface of the membranes, where it was found that for the case of the membranes functionalized with AuNPs-SFw, absorption bands were presented at an approximate wavelength of 530 nm (Figure 4i), while for the membranes functionalized with AgNPs-SFw it was not possible to obtain the characteristic resonance band for silver nanoparticles (Figure 4j), which could indicate that there was a greater deposition of gold nanoparticles than silver on the membrane surface.

Regarding the changes in the functional groups of the membranes with gold or silver nanoparticles, it was evidenced that from the IR absorption spectra for each of the samples (Figure 4k,l) that no significant changes occurred with respect to the treated membranes, in the position of the characteristic bands of the vibrational modes of amides I, II, and III located at wavenumbers between (1623–1619) cm^−1^, (1520–1511) cm^−1^, and (1232–1227) cm^−1^, respectively. When determining the percentage of crystalline and non-crystalline secondary structures, an increase in random coils, turns, and α-helix was found in the functionalized membranes with AuNPs-SF_w_ or AgNPs-SF_w_ compared to the treated membranes without functionalization (Table 3), which is related to the contribution of amorphous secondary structures present in the protein used in the synthesis of the metal nanoparticles.

### 3.5. Linear Sweep Voltammetry

Linear sweep voltammetry consists of the measurement of the faradic current between the WE_G_ and RE as a result of the oxidation-reduction processes of an analyte by applying a change in the electric potential at the working electrode in relation to the fixed potential of the reference electrode [30]. The treated non-functionalized and functionalized with gold nanoparticles or silver membranes were positioned on the WE_G_, and a graphite counter electrode (CE_G_) was used to complete the electrical circuit.

**Figure 5:**
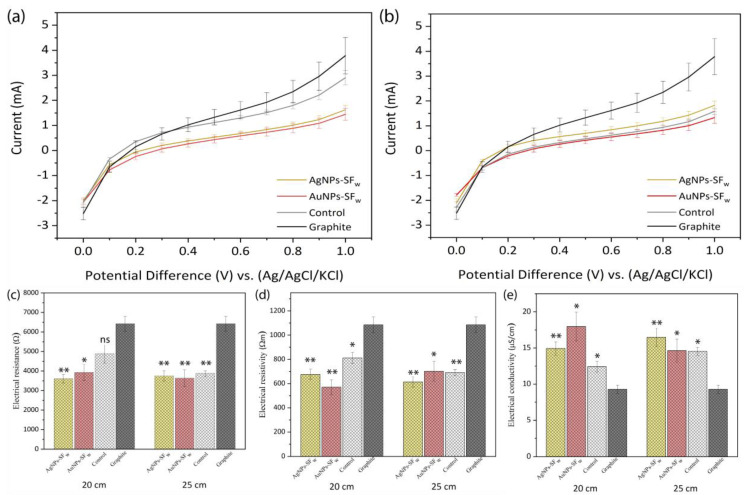
Linear scanning voltammograms were obtained in Hank’s Balanced Salt solution at 100 mV/s. Where (**a**) SF_w_ membranes obtained at 20 cm and functionalized with gold and silver nanoparticles and (**b**) SF_w_ membranes obtained at 25 cm and functionalized with metal nanoparticles. Non-functionalized treated SF_w_ membranes were used as controls. Electrical response of treated SF_w_ membranes at distances of 20 cm and 25 cm with and without functionalization with AuNPs-SF_w_ or AgNPs-SF_w_. Where (**c**) resistivity; (**d**) resistance; (**e**) conductivity. * indicates statistical significance with *p*-values < 0.05 and ** *p*-values < 0.001.

From the results obtained, it was found that, for the treated membranes that were electrospun at 20 cm, the magnitude of the electric current was lower compared to that obtained for the WE_G_. On the other hand, the membranes functionalized with AgNPs-SF_w_ or AuNPs-SF_w_ presented a decrease in the value of the faradic current with respect to that of the non-functionalized membranes and when comparing the functionalized membranes, it was found that those containing AgNPS-SF_w_ exhibited a higher current compared to those functionalized with AuNPs-SF_w_ (Figure 5a). Such behavior is related to the number of species that were oxidized on the surface of the electrode, which is controlled by the standard oxidation potentials (E^0^) of the ionic species of Cl^−^ and H_2_O molecules present in the electrophysiological solution (Equations (1) and (2)). However, the potential increased when approaching the E^0^ of the species present in equilibrium, and the current recorded for the graphite electrode, and the product of the exchange of electrons from the oxidized species towards the surface of this, progressively increased. Furthermore, the absence of oxidation peaks for Cl^−^ and H_2_O in the linear scanning voltammogram is due to the presence of unoxidized species in the vicinity of the electrode surface that can increase the value of the faradic current until reaching a maximum peak and subsequent decrease in current [31].

(1) $$2{\mathrm{Cl}}^{-}{}_{\left(\mathrm{aq}\right)}\to {\mathrm{Cl}}_{2}+2e^{-}(E^{0}≅+1.36\;V)$$

(2) $$2H_{2}O_{\left(l\right)}\to O_{2}{}_{\left(g\right)}+4H^{+}+4e^{-}\left(E^{0}≅+1.23\;V\right)$$

On the other hand, the decrease in the faradic current registered for the SF_w_-treated membranes is related to the lack of electrical coupling between the graphite electrode and the membrane, due to the absence of functional groups present on the surface of the WE_G_, which favor the binding through electrostatic interactions with the functional groups exposed on the surface of the membranes that could facilitate a greater diffusion of electrons generated in oxidation reactions and the favorable response of the electric current [32,33]. Regarding the electrical behavior evidenced in the membranes functionalized with AuNPs-SF_w_ or AgNPs-SF_w_ apart from the oxidation reactions of the Cl^−^ and H_2_O species, two additional reactions related to the oxidation of the metal nanoparticles were presented, which are mediated by the presence of chlorine ions in the solution that leads to the formation of silver chlorides for AgNPs-SF_w_ (Equation (3)) and formation of Au (I) and Au (III) chlorides for AuNPs-SF_w_ (Equations (4) and (5)) [34,35].

(3) $${\mathrm{Ag}}_{\left(s\right)}+{\mathrm{Cl}}^{-}{}_{\left(\mathrm{aq}\right)}\to \mathrm{AgCl}+e^{-}(E^{0}≅+0.22\;V)$$

(4) $${\mathrm{Au}}_{\left(s\right)}+2{\mathrm{Cl}}^{-}{}_{\left(\mathrm{aq}\right)}\to {\mathrm{AuCl}}_{2}{}^{-}+e^{-}(E^{0}≅+1.15\;V)$$

(5) $${\mathrm{Au}}_{\left(s\right)}+4{\mathrm{Cl}}^{-}{}_{\left(\mathrm{aq}\right)}\to {\mathrm{AuCl}}_{4}{}^{-}+3e^{-}(E^{0}≅+1.00\;V)$$

The decrease in the value of the electric current is related to the lack of electrical coupling between the metal nanoparticles and the surface of the electrospun membranes, where the functionalization mediated by the impregnation coating generates a thin film on the material, which does generate modifications in the exposed functional groups, which causes the electrons generated by the oxidation of the gold or silver nanoparticles arranged along the film to not diffuse through the material and reach the WE_G_, generating an increase in the value of the registered current and obtaining an oxidation peak for the case of AgNPs-SF_w_. Moreover, the difference in the faradic current registered for the treated membranes and functionalized with gold or silver nanoparticles correlates with the standard oxidation potential, which is lower for AgNPs-SF_w_ compared to oxidation to Au (III) by AuNPs-SF_w_, which favored the generation of more electrons due to greater oxidation of metallic silver ions and an increase in the faradic current captured by the electrode.

In relation to the membranes at 25 cm (Figure 5b), the electrical behavior was similar to that exhibited by the membranes obtained at 20 cm, where the WE_G_ presented a greater magnitude of the electric current compared to the membranes treated with and without functionalization with metal gold or silver nanoparticles. The SF_w_-treated membrane at 25 cm presented a lower current value compared to that obtained for the membrane at 20 cm, which is related to the fibrillar morphology, where the membranes at 25 cm presented a larger fiber size, which decreased the exposed surface area of the membranes and made it difficult for Cl^−^ ions and/or H_2_O molecules to be oxidized on their surface and generate a greater current of electrons that were diffused to the surface of the WE_G_.

On the other hand, the little variation in the faradic current obtained in the membranes at a distance of 25 cm and functionalized with AuNPs-SF_w_ or AgNPS-SF_w_ compared to the response of the membranes at a distance of 20 cm is associated with the formation of the thin film of nanoparticles on the surface of the membrane, which, as there was no modification in the functionalization procedure, resulted in a slight variation in the number of electrons diffused to the WE_G_ product of the oxidation of AuNPs-SF_w_ and AgNPs-SF_w_ in the presence of Cl^−^ ions.

When coating the graphite electrode with the SF_w_ membranes treated with and without functionalization with the metal nanoparticles, a favorable response was found for each membrane evaluated in the electrochemical cell as a result of the different oxidation reactions involved according to the type of biomaterial of study, which was related to the concentration gradients of the ionic species present on the surface of the WE_G_ and in the electrophysiological solution, which generated variations in the potential described by the Nernst (Equation (6)) [30].

(6) $$E=E^{0}+\frac{0.059}{n}\cdot \log\frac{\prod {\left[Ox\right]}^{n}}{\prod {\left[Red\right]}^{n}}$$

where *n* is the number of electrons transferred, $\prod {\left[Ox\right]}^{n}$ y $\prod {\left[Red\right]}^{n}$ is the product operator of the concentrations of the oxidized and reduced species, raised to their stoichiometric coefficients in the vicinity of the WE_G_.

The results presented a behavior similar to that exhibited by the cardiac action potential, where a reversible change in the membrane potential is generated by means of a bioelectric stimulus in the form of an electric current, produced by the sequential activation of various ionic currents generated by the diffusion of ions through the membrane in favor of its electrochemical gradient. This membrane potential is also described by the Nernst’s Equation, which is mediated by the concentration gradients of the Na^+^, Ca^2+^, Cl^−^ ions, and the K^+^ concentration inside $\left({\left[K^{+}\right]}_{i}\right)$ and outside $\left({\left[K^{+}\right]}_{e}\right)$ of the cell membrane (Equation (7)) [36].

(7) $$E_{K}=-61\cdot \log\frac{{\left[K^{+}\right]}_{i}}{{\left[K^{+}\right]}_{e}}$$

Based on the above, the electrochemical response obtained from SF_w_ electrospun treated membranes and functionalized with AuNPs-SF_w_ or AgNPs-SF_w_ could favor the diffusion of a bioelectric stimulus in the form of an electric current by modifying the resting potential and favoring the cardiac action potential. Furthermore, the functionalized membranes could exhibit favorable behavior when interacting in an in vitro model of functional cardiac cells. Therefore, these could be used as a therapeutic strategy for cardiac tissue engineering as an electroconductive bioactive scaffold.

On the other hand, Figure 5c–e show the electrical resistance, through the derivative of the voltage with respect to the current (dV/dI) at the place where the tangent touched the voltagram at a point, likewise, the resistivity (*ρ*) and conductivity (*σ*) (Equations (8) and (9)) of the treated membranes at 20 cm and 25 cm and functionalized with AuNPs-SF_w_ and AgNPs-SF_w_.

(8) $$\rho =R\cdot \frac{S}{l}$$

(9) $$\sigma =\frac{1}{\rho }$$

where *R* is the resistance in ohms, *S* is the exposed area in m^2^, and *l* is the separation between the working and reference electrode in m.

Considering each material as an individual working electrode evaluated in the electrochemical cell, it was found that the SF_w_ membranes treated with and without functionalization with the metal nanoparticles generated less opposition to the flow of electric current compared to the graphite electrode from the results of resistance, resistivity, and electrical conductivity, which is associated with greater fiber connectivity in the treated membranes and the intrinsic electrical properties of the nanoparticles. Similarly, it could be noted that by increasing the distance from 20 cm to 25 cm, the SF_w_-treated membranes presented an increase in electrical conductivity, which could be related to the arrangement of the fibers and their size, whereas the membranes functionalized with AugNPs-SF_w_ or AgNPs-SF_w_ showed variation in electrical conductivity, which is related to the number of nanoparticles arranged on the surface of the material.

### 3.6. Electrochemical Impedance Spectroscopy

**Figure 6:**
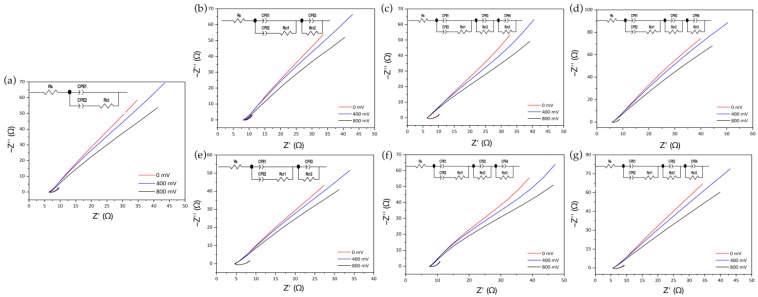
Nyquist plots between Z″ y Z′ for the graphite electrode, treated SFw membranes with and without functionalization in Hank’s Balanced Salt solution at different DC electrical potentials. Where (**a**) graphite electrode; (**b**) treated SFw membrane at 20 cm; (**c**) AgNPs-SFw; (**d**) AuNPs-SFw; (**e**) treated SFw membrane at 25 cm; (**f**) AgNPs-SFw; (**g**) AuNPs-SFw. At the top left of each diagram, the Randles equivalent circuit is presented.

Through electrochemical impedance spectroscopy, it is possible to find the electrochemical behavior of different biomaterials when interacting with the graphite working electrode by applying a small amplitude sinusoidal excitation signal and measuring the response in the form of current, voltage, or another signal of interest [37]. Therefore, impedance measurements were carried out at an amplitude of 86 mV, which emulated the transmembrane potential reached in the electrical depolarization of cardiac cells and at frequency sweeps between 1 × 10^6^ to 0.1 Hz. Figure 6a presents the Argand plots or Nyquist plots for the graphite electrode without interacting with the membrane, where it presented a linear relationship between the imaginary impedance (Z″) with respect to the real impedance (Z′), in which as the direct current electric potential applied to the system increased, a lower slope was presented, indicating that the kinematic reaction is limited by diffusion processes [37,38]

The behavior found for the graphite electrode could be related to the electrical double layer generated when interacting with Hank’s Balanced Salt Solution, which under specific conditions can exhibit capacitive and conductive properties. Concerning to the elements that make up the Randles circuit, the parameter R_s_ is associated with the resistance of the solution, the CPE1 corresponds to the non-ideal capacitive behavior of the electrode, which is strongly dependent on the frequency of the electrical conductivity, and CPE2 is due to the capacitance of the electrical double layer between the polarized WE and Hank’s solution, while R_ct_ represents the resistance to charge transfer between the solution and the electrode surface. The use of CPE constant phase elements instead of capacitors allowed us to achieve a better fit of the experimental data due to the lack of homogeneity in the system associated with the rough or porous surface of the electrode [39,40,41].

Regarding the Nyquist plots obtained for the treated SF_w_ membranes with and without functionalization (Figure 6b–g), a similar behavior to that presented for the graphite electrode was observed, preserving the linear relationship between Z″ and Z′ and the slope value decrease as the DC electric potential increased. Based on these results, the Randles circuits were obtained, finding that the equivalent circuit obtained for the graphite electrode did not allow the adjustment of the experimental results for the treated membranes without or with functionalization. Thus, the circuit was complemented with elements composed of a CPE and R_ct_ in parallel connected in series with the previous circuit, which corresponded to each of the additional bioactive electrochemical layers [42].

**Figure 7:**
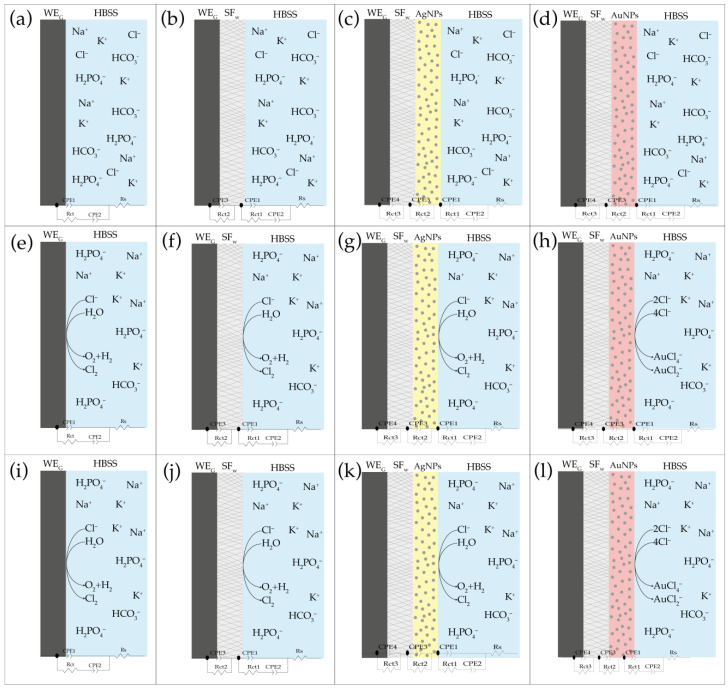
Schematic representation of the electrical behavior for the graphite electrode and the interaction with treated membranes with and without AuNPs-SF_w_ or AgNPs-SF_w_ functionalization, which describe the distribution of impedance components with their respective equivalent circuits to different DC electrical potentials. Where (**a**–**d**) 0 mV; (**e**–**h**) 400 mV; (**i**–**l**) 800 mV.

In Figure 7, an overview of the electrochemical behavior found and its corresponding equivalent circuit is presented, showing that at electric potentials of 0 mV (Figure 7a–d), an electrochemical equilibrium was presented between the ionic solution and working electrode with and without functionalization with the nanoparticles. However, as the electric potential increased to values of 400 mV (Figure 7e–h) and 800 mV (Figure 7i–l), the different oxidation reactions began to occur, which triggered the appearance of constant phase elements in equivalent circuits.

From the results, it was observed that when performing variations in the direct current electric potential, changes were generated in the *C_CPE1_* capacitance parameter and the exponent n corresponding to the constant phase element, which indicates the ability of a material to acquire and store energy in the form of an electric charge and its behavior as a capacitor or resistor, which vary as a result of the different oxidation reactions that occur on the surfaces of the treated membranes with or without functionalization with metal nanoparticles, generating electrons that are stored for short periods of time in the biomaterial or diffused through it [43].

On the other hand, regarding the change in the resistance *R_ct_*_1_ due to the charge transfer between the solution and the surface of the type of membrane with which it interacts, this is strongly influenced by any modification of the electrode surface, finding that for the graphite electrode as electrical potential increases, resistance decreases. The opposite occurs for the different coatings of the working electrode with the treated membranes with or without functionalization with metal nanoparticles, whereas as the potential increased, the resistance increased due to the charge transfer processes. Such behavior could be related to (i) the electrostatic repulsion between the surface charge of both the treated SF_w_ membranes, as well as those functionalized with AuNPs-SF_w_ or AgNPs-SF_w_ with ionic species carrying the same charge; and (ii) steric hindrances generated by structural modifications of the protein and/or by the presence of metal nanoparticles [39].

**Figure 8:**
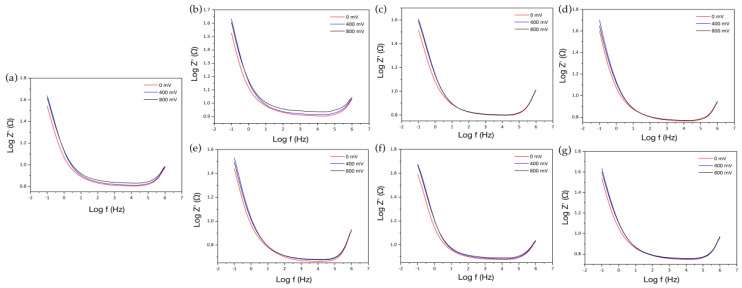
Bode plots between log_10_ |Z| y log_10_f for the graphite electrode, treated SF_w_ membranes with and without functionalization in Hank’s Balanced Salt solution at different DC electrical potentials. Where (**a**) Graphite electrode; (**b**) SF_w_ obtained at 20 cm; (**c**) AgNPS-SF_w_; (**d**) AuNPs-SF_w_; (**e**) SF_w_ obtained at 25 cm; (**f**) AgNPS-SF_w_; (**g**) AuNPs-SF_w_.

To determine the impedance for the different membranes evaluated by EIS, Bode plots were used to evaluate the change in the magnitude of the impedance with respect to the frequency. Finding that both for the graphite electrode (see Figure 8a) and for the treated SF_w_ membranes obtained at distances of 20 cm and 25 cm with their respective surface modifications with AuNPs-SF_w_ or AgNPs-SF_w_ (see Figure 8b–g), it was determined that as the electric potential increased there was an increase in impedance. On the other hand, it was observed that, for the frequency range between 2 × 10^5^ Hz to 100 Hz, an almost constant impedance value was presented, indicating a resistive behavior, while, at values lower than 100 Hz, a linear behavior was presented with a high slope, which could indicate capacitive behavior [44].

To evaluate the change in the impedance value when coating the graphite electrode, the value of the impedance magnitude was determined at a frequency of 1,995 Hz close to a normal heart rate range, determined from 120 beats per minute, equivalent to 2 Hz. The results were not favorable as a function of the frequency close to that of the heart due to the lack of coupling through electrostatic interactions between the surface of the graphite electrode and the SF_w_ membranes treated with and without functionalization (Table 4).

On the other hand, if only the first part of the equivalent circuit [$R_{s}\cdot \left(CPE1\cdot \left(CPE2\cdot R_{ct1}\right)\right)]$ is considered, that is, the parameter R_s_ associated with the resistance of the solution, *CPE*1 corresponding to the non-ideal capacitive behavior of the electrode, *CPE*2 is the capacitance of the electric double layer, and R_ct1_ is the resistance to charge transfer between the solution and the electrode surface, and the impedance is determined at a frequency of 2 Hz using Equations (10)–(14), a favorable impedance behavior is obtained when coating the working electrode with each of the treated membranes and functionalized with metal nanoparticles [45].

(10) $$Z_{Rs}=R_{s}$$

(11) $$Z_{CPE}=\frac{1}{C_{CPE}\cdot {\left(j\cdot \omega \right)}^{n}}$$

(12) $$Z_{CPE1,2}=\frac{e^{-2.528\cdot n}\cdot Cos\left(1.571\cdot n\right)}{C_{CPE1,2}}$$

(13) $$Z_{Rct1}=R_{ct1}$$

(14) $$Z_{T}=R_{s}+\frac{1}{\frac{1}{Z_{CPE1}}+\frac{1}{Z_{CPE2}+Z_{Rct1}}}$$

where *C_CPE_* corresponds to the capacitance in farads and *ω* is the angular frequency (ω=2πf). Equation (13) corresponds to the real part of Equation (12) evaluated at a frequency of 1995 Hz, which was obtained using (Maple^TM^ 18 software, Maplesoft, Waterloo, Canada) [37].

The results showed that the increase in the total impedance values of the circuit $R_{s}\cdot \left(CPE1\cdot \left(CPE2\cdot R_{ct1}\right)\right)$ for the treated SF_w_ membranes, functionalized with AuNPs-SF_w_ or AgNPs-SF_w_ at direct current electric potentials of 400 mV and 800 mV, is related to the formation of Au (I) and Au (II) chloride species and AgCl during the oxidation processes of metal nanoparticles in the presence of Cl^−^ ions, which modify the electrical properties of the composite layer-by-layer and increase the overall impedance of the electrical system.

### 3.7. Cell Viability of Membranes

**Figure 9:**
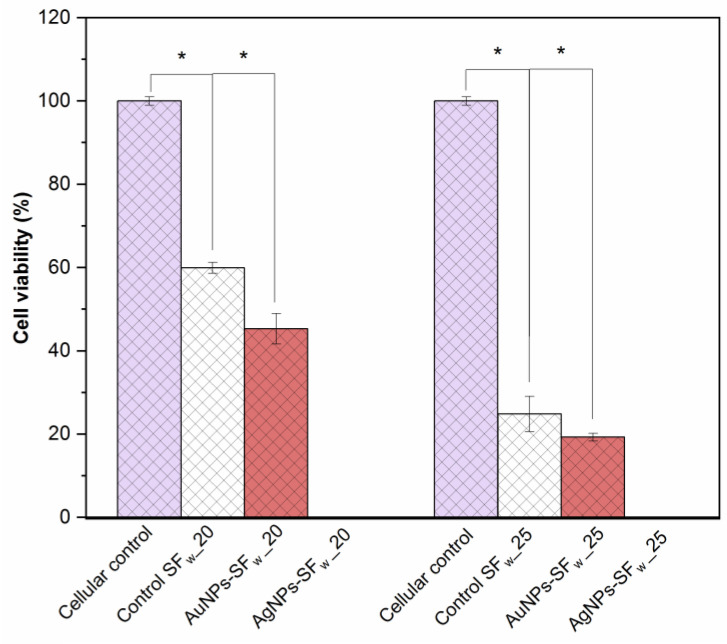
Cell viability of 3T3 fibroblasts on interaction with membranes without or functionalized with the gold or silver nanoparticles. Where _20 represents membranes manufactured at 20 cm and _25 at 25 cm. * indicates statistical significance with *p*-values < 0.05.

The membranes at 20 cm and 25 cm that were not functionalized with nanoparticles presented viability of 60% and 25%, respectively. The membranes at 20 cm and 25 cm functionalized with gold nanoparticles caused viability of the fibroblasts of 45% and 19%, respectively (Figure 9). The results demonstrate that the microarchitecture of the membranes functionalized with gold nanoparticles favors cellular bioavailability and biocompatibility [46].

On the other hand, membranes functionalized with silver nanoparticles do not allow the generation of syncytium cells, and, on the contrary, generated cell toxicity and death. Therefore, this type of membrane could not be used as conductive structures for tissue engineering, due to their oxidative capacity, which causes a limiting barrier in cell–cell communication and could favor cell death processes and induce proinflammatory effects [47].

## 4. Conclusions

Based on the results of our research, it was possible to generate SF_w_/PEO fibrillar membranes, modulating the morphological properties of the electrospun structure without the presence of defects. The treatment with organic solvents such as methanol contributed to these membranes, going from being soluble in aqueous solvents to insoluble in them by a crystalline transition of fibroin macromolecules.

Composed membranes with AuNPs-SF_w_ or AgNPs-SF_w_ were obtained layer-by-layer by impregnation techniques. The resulted membranes showed favorable electrical response with average conductivity values of 16.3 μS/cm (AuNPs-SF_w_), 15.7 μS/cm (AgNPs-SF_w_), and 13.5 μS/cm (Treated SF_w_) compared to 9.3 μS/cm for graphite, with which this type of membrane allowed the diffusion of electrical stimuli behaving as stimulators and modulators of electrical current.

On the other hand, it was found that treated SFw electrospun membranes at distances of 20 cm and 25 cm, coated with AuNPs-SFw or AgNPs-SFw, reduce the impedance values, which could modulate charge transfer and modify the conduction rate of the electrical stimulus when evaluated in an electrophysiological solution.

Furthermore, it was found that electrospun membranes at 20 cm functionalized with AuNPs-SFw generate an increase in electrical conductivity when evaluated in an electrophysiological solution and favor of cell viability. Therefore, it is considered that this type of biomaterial composed of layers could be used as an electroconductive biomaterial for tissue engineering.

## Figures and Tables

**Table 1:** Wavenumbers for the main amide vibrational modes of the pristine SF_w_ spectra, electrospun membranes without and with methanol treatment for 10 min and 15 min.

Sample	Vibrational Modes		
	Amide I (cm^−1^)	Amide II (cm^−1^)	Amide III (cm^−1^)
SF_w_	1635	1515	1230
F50 ^1^d20 ^2^—control	1640	1528	1241
F50d20—10 min	1620	1509	1230
F50d20—15 min	1621	1515	1227
F50d25—control	1640	1528	1241
F50d25—10 min	1621	1513	1230
F50d25—15 min	1620	1511	1229

^1^ FXX—Ratio of SF_w_ used in the solution. ^2^ dXX—Fiber collection distance.

**Table 2:** Secondary structures present in the vibrational mode of amide I for treated and untreated electrospun membranes with methanol for 10 min and 15 min. SF_w_ films were used as control.

Structure Type	SF_w_	SF_w_/PEO (Untreated)		F50d20		F50d25	
		d20	d25	10 min	15 min	10 min	15 min
β-sheets (strong, weak, intra and intermolecular)	28%	18%	12%	56%	64%	46%	55%
β-turns	-	-	-	33%	2%	-	32%
Side chains (Tyr)	5%	2%	2%	7%	8%	7%	7%
Random coils	24%	45%	51%	-	8%	37%	-
α-helix	39%	-	-	-	18%	-	-
turns	4%	35%	35%	4%	1%	10%	5%

**Table 3:** Secondary structures present in the vibrational mode of amide I for the treated membranes and functionalized with AuNPs-SF_w_ and AgNPs-SF_w_ for 4 h and 24 h. Non-functionalized treated SF_w_ membranes were used as controls.

Structure Type	SF_w_ (Control)	AuNPs-SF_w_				AgNPs-SF_w_			
		UW		W		UW		W	
		4 h	24 h	4 h	24 h	4 h	24 h	4 h	24 h
β-sheets (strong, weak, intra and intermolecular)	55%	52%	60%	52%	55%	48%	47%	46%	46%
β-turns	32%	0%	2%	38%	1%	-	-	-	-
Side chains (Tyr)	7%	4%	8%	5%	2%	5%	4%	4%	4%
Random coils	-	4%	11%	-	26%	39%	34%	37%	38%
α-helix	-	35%	12%	-	1%	-	-	-	-
Turns	5%	4%	8%	5%	16%	8%	14%	13%	12%

Where UW—unwashed and W—washed.

**Table 4:** Impedance values obtained at different DC electric potentials for the different membranes evaluated by EIS obtained at a frequency of 2 Hz.

Sample	Electric Potential		
	0 mV	400 mV	800 mV
Graphite	9.80	10.72	10.95
Treated SF_w_ at 20 cm	11.39	12.23	12.54
AuNPs-SF_w_	9.73	10.57	10.43
AgNPs-SF_w_	10.01	10.89	10.82
Treatred SF_w_ at 25 cm	7.80	8.49	8.37
AuNPs-SF_w_	9.36	10.18	10.12
AgNPs-SF_w_	11.47	12.58	12.65

## Data Availability

The data presented in this study are available on request from the corresponding author.
